# Thymidylate synthase polymorphisms in genomic DNA as clinical outcome predictors in a European population of advanced non-small cell lung cancer patients receiving pemetrexed

**DOI:** 10.1186/1479-5876-12-98

**Published:** 2014-04-14

**Authors:** Estefanía Arévalo, Eduardo Castañón, Inés López, Josefa Salgado, Víctor Collado, Marta Santisteban, María Rodríguez-Ruiz, Patricia Martín, Leire Zubiri, Ana Patiño-García, Christian Rolfo, Ignacio Gil-Bazo

**Affiliations:** 1 Department of Oncology, Clínica Universidad de Navarra, 31008 Pamplona, Spain; 2 Division of Oncology, Center for Applied Medical Research (CIMA), 31008 Pamplona, Spain; 3 Laboratory of Clinical Genetics, Clínica Universidad de Navarra, 31008 Pamplona, Spain; 4 Department of Radiation Oncology, Clínica Universidad de Navarra, 31008 Pamplona, Spain; 5 Oncology Department, Antwerp University Hospital UZA, 2650 Edegem, Belgium

**Keywords:** Thymidylate synthase, Polymorphisms, Epidermal growth factor receptor, Predictive factors, Prognostic factors, Non-small cell lung cancer

## Abstract

**Background:**

We studied whether thymidylate synthase (*TS*) genotype has an independent prognostic/predictive impact on a European population of advanced non-small cell lung cancer (NSCLC) patients receiving pemetrexed.

**Methods:**

Twenty-five patients treated with pemetrexed-based regimens were included. Genomic DNA was isolated prior to treatment. The variable number of tandem repeat (VNTR) polymorphisms, the G > C single nucleotide polymorphisms (SNP) and the TS 6-bp insertion/deletion (6/6) in the 3′ untranslated region (UTR) polymorphisms were analyzed and correlated with overall response rate (ORR), progression-free survival (PFS), overall-survival (OS) and toxicity.

**Results:**

The genotype +6/+6 predicted a higher ORR among active/former smokers compared to +6/-6 genotype (100% vs. 50%; p = 0.085). Overall, the 3R/3R genotype predicted a higher ORR (100%) over the rest VNTR polymorphisms (p = 0.055). The presence of 3R/3R genotype significantly correlated with a superior ORR in patients without *EGFR* activating mutations (100%) compared to 2R/2R, 2R/3R and 3R/4R genotype (77.8%, 33.3% and 0% respectively; p = 0.017). After a median follow-up of 21 months, a trend towards a better PFS, although not significant, was found among subjects showing 3R/3R polymorphisms (p = 0.089). A significantly superior OS was found in patients showing 3R/3R genotype rather than other VNTR polymorphisms (p = 0.019). No significant correlation with the toxicity was observed.

**Conclusion:**

In our series, 3R/3R polymorphism correlated with a superior OS. Also, this polymorphism, when associated to wild type *EGFR,* was related to a higher ORR to pemetrexed. Toxicity was not significantly correlated with a specific TS genotype.

## Background

Lung cancer represents the most frequent cause of cancer deaths. More than 225,000 new cases were diagnosed during 2012 only in the United States of America, accounting for approximately 160,000 annual deaths [1,2]. More than 50% of the patients diagnosed with non-small cell lung cancer (NSCLC) present advanced disease (stage III and IV) at onset. The most common histology is adenocarcinoma representing approximately 80% of all cases [3].

Pemetrexed, is a multitargeted antifolate drug and one of the latest active drugs against NSCLC [4] approved for the first-line [5] (in combination with cisplatin) [6] and second-line treatment (monotherapy) of patients with non-squamous histology [7]. More recently, pemetrexed gained approval for its use as a single-agent maintenance therapy [8] after response/stabilization to four cycles of a platinum doublet with or without pemetrexed.

Thymidylate synthase (*TS*) is the main biological target of antifolate drugs such as pemetrexed or 5-fluorouracil. Different studies have evaluated the correlation between tumor TS expression and *TS* genotype and the prognosis of patients with different cancer types treated with antifolates [9-11]. In NSCLC, constitutive expression of TS is lower in tumors with adenocarcinoma histology than among those with squamous differentiation [12]. This finding could possibly explain the higher efficacy of the drug among non-squamous histology patients. The potential predictive role of *TS* polymorphisms in NSCLC has never been studied in a European population. In addition, how differential *TS* genotypes may impact on the outcome of patients depending on their smoking status or with Epidermal Growth Factor Receptor *(EGFR)* activating mutations tumors is to be determined.

Finally, although the toxicity profile described in most patients receiving pemetrexed in combination or as a single agent is usually favorable, there are several reported cases of fundamentally dermatological, hematological and potentially serious renal toxicities, even when the recommended vitamin prophylaxis guidelines have been followed [13-15]. Nonetheless, the tumor TS levels or its polymorphisms in each patient could explain these cases of severe toxicity, as it has been suggested in other neoplasms treated with other antifolate drugs [9]. The potential association between different *TS* genotypes and the toxicity experienced by a European population of patients with NSCLC receiving pemetrexed is also to be studied.

Three different types of polymorphisms have been described in the *TS* gene. In the gene promoter, there is a variable number of tandem repeats (VNTR) of 28 pb in the 5′- region. Thus, cases of two or three repetitions of this tandem *TS* gene promoter enhancer region (TSER) have been described [16]. A second type of polymorphism consists in a change in a single nucleotide (G > C), in one of the sequences of the repetition comprising a single nucleotide polymorphism (SNP) [16]. A third modality of polymorphisms consists in the deletion or insertion of 6 pair of bases (bp) in the 3′-UTR region (untranslated region) [16].

In summary, the potential usefulness of *TS* genotype as an independent prognostic factor or predictor of response to pemetrexed-based regimens in a European NSCLC population has not been studied. Similarly, no clear evidence is available about the potential correlation between the different *TS* genotypes and the toxicity experienced by those patients.

Therefore we decided to investigate the three known polymorphisms of *TS* gene, and their correlation with objective response rate (ORR), progression-free survival (PFS) and overall survival (OS), as well as toxicity in European patients with advanced NSCLC treated with pemetrexed-based regimens.

## Methods

### Patients and samples

Overall, 25 consecutive stage III-IV NSCLC patients treated at a single institution, from which peripheral blood samples were available, were analyzed. All of them received pemetrexed-based regimens in the first, second or third line settings, according to our institutional therapeutic protocols.

After the informed consent was obtained from all patients, 10 ml of peripheral blood samples were collected before the administration of the first cycle of a pemetrexed-containing regimen. Blood samples were stored at the Biobank of the University of Navarra and were processed following standard operating procedures approved by the Ethical and Scientific Committees.

Tumor ORR to the treatment was assessed using computerized tomography (CT) scans every two pemetrexed-based chemotherapy cycles and categorized according to the Response Evaluation Criteria In Solid Tumors (RECIST) v1.1, as per institutional protocol.

The toxicities recorded during pemetrexed-based treatment were graded according to the Common Terminology Criteria for Adverse Events (CTCAE) version 4.0.

### TS enhancer region genotyping analysis

The genomic DNA was extracted from the peripheral leucocytes. The genotypes of the TSER (VNTR) and SNP were determined by polymerase chain reaction (PCR). The variable number tandem repeat (VNTR) of 28 bp polymorphism and the G → C SNP in the first and second repeat were analyzed. A DNA fragment was amplified using previously described PCR conditions and primers [17], and directly sequenced using an ABI PRISM 3100 Genetic Analyzer (Applied Biosystems, Foster City, CA, USA). The forward primer 5′-CGTGGCTCCTGCGTTTCC-3′ and the reverse primer 3′-GAGCCGGCCACAGGCAT-5′ were used. A modification of conventional conditions was necessary. PCR was performed in a reaction mixture with dNTP: 0.35 μl, Buffer: 0.25 μl, MgCl2: 17.5 μl, Tap polymerase: 0.5 μl, H2O: 18 μl, primers 0.1 + 0.1 μl, DMSO: 1.25 μl and DNA: 2 μl. The cycling conditions were denaturation 95°C for 10 minutes, and 30 cycles at 95°C for one minute, then at 64°C for one minute, and 72°C one minute, and finally, seven minutes at 72 ºC. Aliquots of amplified fragments were separated on a 3% agarose gel and the TS VNTR genotype was determined, staining 2R (210 base pairs; bp) and 3R (238 bp) alleles. After that we performed a PCR-restriction fragment length polymorphisms (RFLP), by Hae III digestion. The mixture was PCR product: 10 μl, H2O: 7 μl, Buffer 2 μl, and Hae III: 1 μl. After that we incubated the mixture at 37°C overnight. Aliquots of digested fragments were separated on 12% acrylamide gel and the SNP genotype was determined. The digestion of fragments showed the different genotypes 2RGC: 66, 47, 46, 44 and 7 bp, 2RCC: 113, 46, 44 and 7 bp, 3RGGCC (3RG): 66, 47, 46, 44, 28 and 7 bp, 3RGCC (3RC): 94, 47, 46, 44 and 7 bp.

### TS 3′UTR region genotyping analysis

The 3′UTR polymorphisms were analyzed by Restriction Fragment Length Polymorphism (RFLP). A fragment containing the 6 bp deletion/insertion was amplified using the reverse primer 5′-CAGATAAGTGGCAGTACAGA-3′and the forward primer 3′-CAAATCTGAGGGAGCTGAGT-5′ in 10 ul of reaction mixture with dNTP: 4 μl, Buffer: 5 μl, MgCl2: 4 μl, Tap polymerase: 0.5 μl, H2O: 26.5 μl, primers 4 + 4 μl and DNA: 2 μl. The cycling conditions were denaturation 95°C for 10 minutes, and 35 cycles at 95°C for 30 minutes, then at 57°C for 30 minutes, and 72°C one minute, and finally, seven minutes at 72°C. The fragments were amplified on 2% agarose gel. Afterwards the products were digested with Dra I and the mixture of PCR product: 20 μl, BSA 10%: 0.5 μl, Buffer: 5 μl, H2O: 23.5 μl and Dra I: 1 μl. Posteriorly, the product was incubated one hour at 37°C. The final digested product was separated in a 3% agarose gel. The different genotypes were deletion 6 bp/insertion 6 bp, insertion 6 bp/insertion 6 bp and deletion 6 bp/deletion 6 bp. The expected fragment sizes by genotyping were deletion 6 bp/insertion 6 bp: 148, 142, 88 and 60 bp, insertion 6 bp/insertion 6 bp: 88 and 60 bp, and deletion 6 bp/deletion 6 bp: 142 bp. We repeated the PCR three times to ensure final results.

### *EGFR* mutations analysis

As per institutional protocol, all patients with advanced NSCLC were tested for *EGFR* activating mutations before treatment initiation. In brief, after having the samples fixed in alcohol and stained by Papanicolau stain, DNA was extracted and amplified by PCR technique, using *EGFR* gene exons 18, 19, 20 and 21 specific primers. ABI PRISM® 310 Genetic Analyzer equipment was used for the analysis of the sequencing reactions with both forward and reverse primers.

### Statistical analysis

Fisher’s exact test was used to investigate the correlation between each genotype and the response to the treatment and the toxicity presented. Kaplan-Meier curves and log-rank test or Tarone-Ware test, when indicated, were calculated to correlate each genotype with the survival outcomes (PFS and OS). For the subgroup analysis, *EGFR* mutation status and smoking history were considered in order to analyze potential differences in clinical outcome measures (ORR, PFS and OS).

The SPSS 15.0 software (SPSS, Inc., Chicago, IL) was employed to perform the statistical analysis.

## Results

### Patients’ characteristics and treatment

The clinical and pathological characteristics of the patients included are summarized in Table 1. In brief, our cohort was mainly composed by males with a median age of 59 years and a past smoking history showing good performance status. Most of the patients showed adenocarcinoma histology (88%) and showed distant metastasis (M1) at onset (72%). Most of the patients received a pemetrexed-based regimen in first line (84%). After a median follow up of 21 months, 80% of patients have already progressed and 52% of them have died due to disease progression (Table 1).

**Table 1:** Patients’ characteristics

	**N pts**	**%**
Gender		
Female	11	44
Male	14	56
Age		
<60	13	52
> r =60	12	48
ECOG		
0	9	36
1	15	60
2	1	4
Tobacco		
Current smoker	4	16
Never smoker	7	28
Former smoker	14	56
Histology		
Adenocarcinoma	22	88
Adenocarcinoma poorly differentiated	2	8
Adeno-squamous	1	4
T		
T1-2	12	48
T3-4	13	52
N		
N0	6	24
N+	19	76
M		
M0	7	28
M1	18	72
Lung metastases		
Presence	7	28
Absence	18	72
Liver metastases		
Presence	2	8
Absence	23	92
Bone metastases		
Presence	10	40
Absence	15	60
Brain metastases		
Presence	8	32
Absence	17	68
*EGFR*		
Wild type	23	92
Mutant	1	4
Unknown	1	4
Line of treatment		
First/Induction (stage III)	2	8
First	21	84
Second	1	4
Third	1	4
Response		
Response	18	72
Progression + Stabilization	7	28
Maintenance		
No maintenance	18	72
Maintenance	7	28
Progression		
Not progressed	6	24
Progressed	19	76
Clinical status		
Alive	12	48
Dead	13	52

Eastern Cooperative Oncology Group (ECOG). Epidermal Growth Factor Receptor (EGFR).

In addition, in 8 out of the 18 subjects showing multiple brain metastases at onset, conventional whole-brain radiotherapy (300 cGy) was administered between first and second chemotherapy cycles, following our institutional treatment guidelines.

Finally, 4 out of the 7 patients showing no distant metastases at onset responded to the pemetrexed-based induction chemotherapy. As per institutional protocol, all four subjects underwent a 3-D conformal radiotherapy program with concurrent chemotherapy, as previously published [18].

### Correlation between ORR to the treatment and polymorphisms

We studied the potential correlation between the different polymorphisms observed and the response to the treatment obtained (Table 2). For this purpose, any kind of radiological response (complete or partial response), was compared to no response to the treatment (disease stabilization or progression). The presence of 3R/3R polymorphism seemed to predict a higher ORR (100%), compared to the rest of the genotypes with a trend toward statistical significance (p = 0.055). In the subgroup analysis, a significantly higher ORR to pemetrexed for wild-type EGFR patients showing a 3R/3R genotype (100%) compared to the 2R/2R (77.8%), 2R/3R (33.3%) and 3R/4R (0%) was observed (p = 0.017).

**Table 2:** Overall response rate to the treatment and polymorphisms observed

**Global distribution of polymorphisms (Pol)**	**Response N (%)**	**Stabilization or progression N (%)**	**p value**
VNTR			
2R/2R	7 (77.8)	2 (22.2)	0.055
3R/3R	7 (100)	0 (0)	
2R/3R	4 (50)	4 (50)	
3R/4R	0 (0)	1 (100)	
Pol VNTR (Subanalysis by *EGFR* status; group of native *EGFR*-patients)			
2R/2R	7 (77.8)	2 (22.2)	0.017
3R/3R	7 (100)	0 (0)	
2R/3R	2 (33.3)	4 (66.7)	
3R/4R	0 (0)	1 (100)	
Global distribution of SNP			
Absence	6 (85.7)	1 (14.3)	0.626
Presence	12 (66.7)	6 (33.3)	
Global distribution of polymorphisms in 3′-UTR			
+6/+6	10 (83.3)	2 (16.7)	0.234
+6/-6	6 (54.5)	5 (45.5)	
-6/-6	2 (100)	0 (0)	
Pol 3′-UTR (Subanalysis by smoking habit stratification; group of active and former smokers)			
+6/+6	8 (100)	0 (0)	0.085
+6/-6	4 (50)	4 (50)	
-6/-6	2 (100)	0 (0)	

No statistically significant differences were observed comparing the presence and the absence of a SNP G > C as shown in Table 2.

Overall, a non-significant correlation between the different 3′-UTR polymorphisms and the ORR was observed. However, the genotype +6/+6 seemed to predict a higher ORR among active/former smokers (A/FS) compared to +6/-6 (100% vs. 50%; p = 0.085).

### Correlation between PFS and polymorphisms

**Figure 1:**
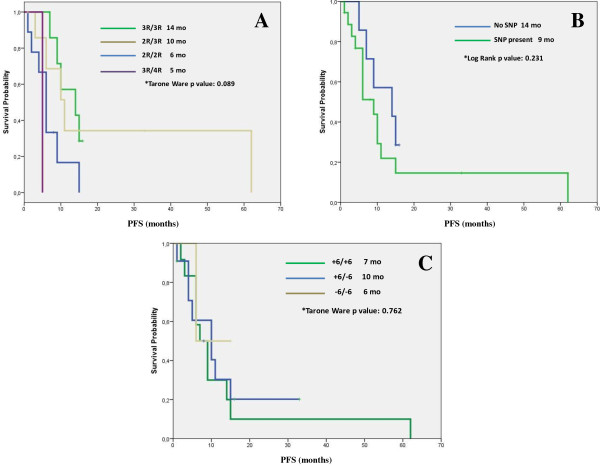
**Kaplan-Meier curves for progression-free survival (PFS) in months (mo) associated with the different TS polymorphisms. A**: TSER genotypes. **B**: Presence or absence of SNP. **C**: 3´UTR genotypes.

Regarding TSER polymorphisms we found a trend toward statistical significance (p = 0.089) in the differences in PFS observed among the different genotypes in favor of the 3R/3R genotype, (Figure 1A).

In the case of the absence or presence of a SNP at the third repetition (3R allele), we observed a non-significant increased PFS in the subgroup of patients showing an absence of SNP (Figure 1B).

Finally, no significant correlations regarding the 3′UTR genotypes and PFS were observed, (Figure 1C).

### Correlation between OS and polymorphisms

**Figure 2:**
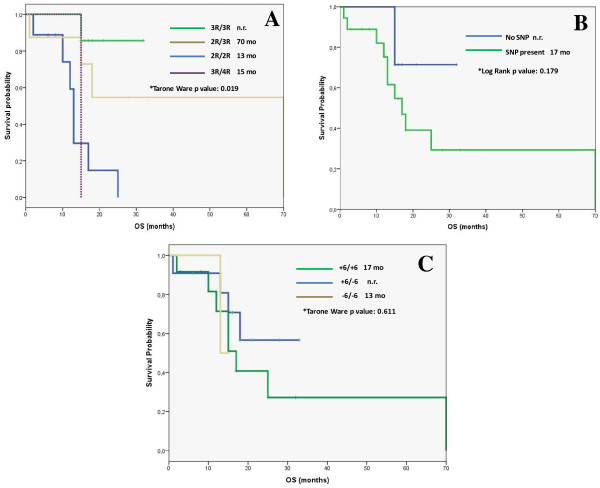
**Kaplan-Meier curves for overall survival (OS) in months (mo) associated with the different TS polymorphisms. A**: TSER genotypes. **B**: Presence or absence of SNP. **C**: 3´UTR genotypes.

In this cohort, we found a significant correlation between TSER polymorphisms and OS (Figure 2A). The median OS was not reached for 3R/3R genotype patients, whereas 2R/3R genotype subjects showed a 70 m OS followed by 3R/4R and 2R/2R genotypes with a median OS of 15 m and 13 m, respectively (p = 0.019), (Figure 2A).

No significant differences in OS were observed with regards to the presence/absence of SNP (Figure 2B) or regarding the 3′-UTR polymorphisms (Figure 2C).

### Correlation between toxicity and polymorphisms

The most frequent toxicity was grade (G)1 anemia (28%) and nausea (20%) and G2 leucopenia (40%). The most commom G3-4 toxicities were leucopenia (16%), asthenia (8%), anemia (4%), neutropenia (4%) and dyspnea (4%). Overall, we found no significant correlations between the toxicity profiles experienced by the patients and the different *TS* genotypes (Table 3).

**Table 3:** Correlation between grades of toxicity and different genotypes

**Global distribution polymorphisms (Pol)**	**No toxicity**	**Grade 1-2**	**Grade 3-4**	**p value**
VNTR polymorphisms				
2R/2R	2 (22.2)	4 (44.2)	3 (33.4)	0.545
3R/3R	2 (25)	5 (75)	0 (0)	
2R/3R	2 (25)	4 (50)	2 (25)	
3R/4R	1 (100)	0 (0)	0 (0)	
SNP polymorphisms				
Absence	3 (42.9)	4 (57.1)	0 (0)	0.3
Presence	4 (22.2)	9 (50)	5 (27.8)	
3′-UTR polymorphisms				
+6/+6	3 (25)	6 (50)	3 (25)	> 0.05
+6/-6	3 (27.3)	6 (54.5)	2 (18.2)	
-6/-6	1 (50)	1 (50)	0 (0)	

## Discussion

Pemetrexed, a multitargeted antifolate drug, is essential for the first and second-line as well as maintenance treatment of NSCLC patients with non-squamous histology [6]. TS is the main biological target of pemetrexed. Some studies have suggested that TS expression could be a predictive factor of response in NSCLC [19]. Moreover, some VNTR genotypes have been associated with TS expression and activity in other tumor types such as colorectal cancer [17].

In NSCLC patients, a correlation between different genotypes and the TS protein expression has been shown [20]. Shintani et al. [20] also confirmed that the TS mRNA levels were significantly higher in lung cancer tissues with the 3R/3R genotype as compared to those with the 2R/2R genotype.

Nonetheless, definitive studies addressing the correlation of the different genotypes of *TS* in circulating genomic DNA with response to the treatment, PFS or OS in pemetrexed-treated NSCLC European patients are lacking. The potential influence of the *EGFR* status on those polymorphisms and their correlation with clinical outcome after pemetrexed-based treatment is also unexplored.

A recent study by Hu et al. [21] investigated the different *TS* polymorphisms in genomic DNA of 90 Asian NSCLC patients. In contrast with our findings, no specific genotype regarding the TSER or 3′-UTR polymorphisms studied seemed to correlate with a significant difference in ORR, PFS or OS. This could be explained by substantial clinical differences between both populations. Our cohort was constituted by Caucasian patients compared to the Asian population studied by Hu et al. In addition, our patients were mostly current or former smokers (72%) compared to the Asian population that showed 62% of never smokers. Also in our cohort, the subjects mainly received pemetrexed-based chemotherapy as a first line regimen (92%), whereas the cohort studied by Hu et al. [21] was treated with pemetrexed as a second or further line in 62.2% of the cases. These remarkable differences in basic clinical characteristics, and in particular the ethnicity, between both cohorts are probably also explaining the differences observed in the 3′-UTR genotype frequency between our population and the one studied by Hu et al. In our cohort, +6 bp/+6 bp, +6 bp/-6 bp and -6 bp/-6 bp genotypes were found in 48%, 44% and 8% of the cases, respectively. In contrast, 7.8%, 47.8% and 44.4% were respectively found in the population studied by Hu et al. [21]. In a previous analysis performed on another Caucasian NSCLC population evaluated at the M.D. Anderson Cancer Center [22], a similar proportion of 3′-UTR genotypes according to our findings was observed (49.2% of +6 bp/+6 bp, 42.4% of +6 bp/-6 bp and 8.4% of -6 bp/-6 bp). Additionally, the low prevalence of the +6 bp/+6 bp genotype in an Asian population compared to our cohort may be confirmed by a recent study in which from 106 Asian NSCLC patients investigated, none of them showed a +6 bp/+6 bp genotype in genomic circulating DNA [23]. Nontheless, in this latter study [23] a significantly higher ORR was observed among patients showing a -6 bp/-6 bp 3′-UTR genotype compared to the ORR reported for patients presenting a -6 bp/+6 bp polymorphism (32.2% vs 12.7%; p = 0.008). Accordingly, in our cohort, a higher ORR in patients showing a -6 bp/-6 bp genotype compared to those presenting a -6 bp/+6 bp polymorphism was also observed (100% vs. 54.5%). However, the statistical significance was not reached probably due to the relatively low number of patients included in our analysis. Interestingly enough, in the subgroup analysis of our data, the +6 bp/+6 bp genotype seemed to predict a higher ORR only among active/former smokers compared to +6 bp/-6 bp (100% vs. 50%; p = 0.085). This novel observation, if validated in future studies, could be relevant for selecting specific drugs for each patient in a second or third line setting.

With regards to the TSER polymorphisms, the presence of a 3R/3R polymorphism seemed to predict a higher ORR with a clear trend toward statistical significance (p = 0.055). Moreover, that difference was even greater and statistically significant benefiting the subpopulation of wild-type *EGFR* patients. To our knowledge, this is the first time that such observation has been made. An interesting preclinical study by Giovannetti et al. [24] investigated the activity profile of a combination therapy against NSCLC cell lines with different genotypes with erlotinib and pemetrexed. Remarkably, pemetrexed increased EGFR phosphorylation and reduced Akt phosphorylation. Additionally, erlotinib significantly reduced TS expression and activity. Thus, when erlotinib and pemetrexed were combined, a strong synergism in all NSCLC cells, regardless of their genetic signature, was observed. This potential crosstalk between the EGFR signaling pathway and the TS expression and activity could in part explain our novel findings showing a significantly higher ORR to pemetrexed in those wild-type *EGFR* patients harboring a 3R/3R polymorphism. However, none of the previous studies have described the *EGFR* status of the patients analyzed and how that status impacted on the ORR to pemetrexed for certain *TS* polymorphisms.

In terms of survival, in the present series after a median follow-up of 21 months, PFS was superior for those patients showing a 3R/3R genotype with a trend toward statistical significance, as expected considering the higher ORR observed for patients with the same TSER genotype. The relatively low statistical power of our clinical cohort may be accounting for the lack of a full statistical significance observed.

Regarding OS, the advantage in PFS observed in patients showing the 3R/3R genotype translated into a significantly higher median OS in patients with the same polymorphism compared to the rest. Conversely, in the study by Hu et al. [21] no specific genotype was significantly correlated with a superior PFS or OS. As aforementioned, the dramatic differences in the population’s characteristics between both series might possibly explain this discordance. In our study, conversely to the observations made by Wang et al. [23] a significantly superior PFS and OS in patients with the -6 bp/-6 bp 3′-UTR genotype has not been confirmed. Most probably, this is also due to the differences in the genotype distribution among populations with markedly different ethnicity and epidemiological characteristics.

Finally, in accordance with Wang et al. [23], the toxicity profile was not significantly correlated with any *TS* genotype in our series.

As aforementioned, this study has some limitations due to its retrospective nature and the low number of patients investigated. Both could be responsible for a low statistical power that may impair our ability to find significant differences between subgroups of patients.

## Conclusions

For the first time in a European population of NSCLC patients receiving pemetrexed, the presence of the TSER 3R/3R polymorphism significantly correlated with a superior OS. Moreover, this same polymorphism, when associated to wild-type *EGFR*, was correlated with a higher ORR to pemetrexed. The presence of the +6/-6 bp 3′-UTR genotype among active or former smokers was correlated to a higher ORR showing a trend toward statistical significance. Finally, pemetrexed-induced toxicity was not significantly correlated with a specific *TS* genotype.

These novel data warrant further investigation in larger prospective series and may help to patient’s selection if finally validated.

## Abbreviations

NSCLC: Non-small cell lung cancer; TS: Thymidylate synthase; EGFR: Epidermal Growth Factor Receptor; VNTR: Variable number of tandem repeats; TSER: Thymidylate Synthase gene promoter enhancer region; SNP: Single nucleotide polymorphism; ORR: Overall response rate; PFS: Progression-free survival; OS: Overall survival; CT: Computerized tomography; RECIST: Response Evaluation Criteria in Solid Tumors; CTCAE: Common Terminology Criteria for Adverse Events; PCR: Polymerase chain reaction; RFLP: Restriction fragment length polymorphism; A/FS: Active/former smokers.

## Competing interests

The authors declare that they have no competing interests.

## Authors’ contributions

EA participated in the design of the study, contributed to the patients’ identification and medical history charts revision and performed samples’ processing and statistical analyses as well as manuscript’s drafting. EC participated in the design of the study, contributed to the patients’ identification and medical history charts revision and performed samples’ processing and statistical analyses as well as manuscript’s drafting. IL carried out samples’ processing and laboratory analysis. JS participated in the design of the study and contributed to the laboratory analysis. VC contributed to samples’ processing, laboratory analysis and paper drafting. MS contributed to results´ interpretation and manuscript´s drafting. MRR contributed to drafting the manuscript and revising it critically for important intellectual content. PM aided to conceive the study, and participated in its design and helped to draft the manuscript. LZ contributed to drafting the manuscript and revising it critically for important intellectual content. APG participated in the design of the study and contributed to the laboratory analysis. CR contributed to drafting the manuscript and revising it critically for important intellectual content. IGB conceived the study and its design, contributed to patients’ selection, drafted the manuscript and gave final approval. All authors read and approved the final version of the manuscript.

## References

[B1] Siegel R Naishadham D Jemal A Cancer statistics, 2012 CA Cancer J Clin 2012 62 10 29 10.3322/caac.20138 22237781

[B2] Sánchez MJ Payer T de Angelis R Larrañaga N Capocaccia R Martinez C CIBERESP Working Group Cancer incidence and mortality in Spain: estimates and projections for the period 1981–2012 Ann Oncol 2010 21 Suppl 3 iii30 iii36 20427358 10.1093/annonc/mdq090

[B3] Brambilla E Travis WD Colby TV Corrin B Shimosato Y The new World Health Organization classification of lung tumours Eur Respir J 2001 18 1059 1068 10.1183/09031936.01.00275301 11829087

[B4] Goffin J Lacchetti C Ellis PM Ung YC Evans WK Lung cancer disease site group of cancer care Ontario’s program in evidence-based care. First-line systemic chemotherapy in the treatment of advanced non-small cell lung cancer: a systematic review J Thorac Oncol 2010 5 260 274 10.1097/JTO.0b013e3181c6f035 20101151

[B5] Cohen MH Johnson JR Wang YC Sridhara R Pazdur R FDA drug approval summary: pemetrexed for injection (Alimta) for the treatment of non-small cell lung cancer Oncologist 2005 10 363 368 10.1634/theoncologist.10-6-363 15967829

[B6] Scagliotti GV Parikh P von Pawel J Biesma B Vansteenkiste J Manegold C Serwatowski P Gatzemeier U Digumarti R Zukin M Lee JS Mellemgaard A Park K Patil S Rolski J Goksel T de Marinis F Simms L Sugarman KP Gandara D Phase III study comparing cisplatin plus gemcitabine with cisplatin plus pemetrexed in chemotherapy-naive patients with advanced-stage non-small-cell lung cancer J Clin Oncol 2008 26 3543 3551 10.1200/JCO.2007.15.0375 18506025

[B7] Hanna N Shepherd FA Fossella FV Pereira JR De Marinis F von Pawel J Gatzemeier U Tsao TC Pless M Muller T Lim HL Desch C Szondy K Gervais R Shaharyar Manegold C Paul S Paoletti P Einhorn L Bunn PA Jr Randomized phase III trial of pemetrexed versus docetaxel in patients with non-small-cell lung cancer previously treated with chemotherapy J Clin Oncol 2004 22 1589 1597 10.1200/JCO.2004.08.163 15117980

[B8] Cohen MH Cortazar P Justice R Pazdur R Approval summary: pemetrexed maintenance therapy of advanced/metastatic nonsquamous, non-small cell lung cancer (NSCLC) Oncologist 2010 15 1352 1358 10.1634/theoncologist.2010-0224 21148615 PMC3227931

[B9] Lecomte T Ferraz JM Zinzindohoué F Loriot MA Tregouet DA Landi B Berger A Cugnenc PH Jian R Beaune P Laurent-Puig P Thymidylate synthase gene polymorphism predicts toxicity in colorectal cancer patients receiving 5-fluorouracil-based chemotherapy Clin Cancer Res 2004 10 5880 5888 10.1158/1078-0432.CCR-04-0169 15355920

[B10] Vignoli M Nobili S Napoli C Putignano AL Morganti M Papi L Valanzano R Cianchi F Tonelli F Mazzei T Mini E Genuardi M Thymidylate synthase expression and genotype have no major impact on the clinical outcome of colorectal cancer patients treated with 5-fluorouracil Pharmacol Res 2011 64 242 248 10.1016/j.phrs.2011.04.006 21536130

[B11] Zucali PA Giovannetti E Destro A Mencoboni M Ceresoli GL Gianoncelli L Lorenzi E De Vincenzo F Simonelli M Perrino M Bruzzone A Thunnissen E Tunesi G Giordano L Roncalli M Peters GJ Santoro A Thymidylate synthase and excision repair-cross-complementing group-1 as predictors of responsiveness in mesothelioma patients treated with pemetrexed-carboplatin Clin Cancer Res 2011 17 2581 2590 10.1158/1078-0432.CCR-10-2873 21262916

[B12] Tanaka F Wada H Fukui Y Fukushima M Thymidylate synthase (TS) gene expression in primary lung cancer patients: a large-scale study in Japanese population Ann Oncol 2011 22 1791 1797 10.1093/annonc/mdq730 21321092

[B13] Bosch-Barrera J Gaztañaga M Ceballos J Pérez-Gracia JL López-Picazo JM García-Foncillas J Ferrer M Sanz ML Pretel M Idoate MA Gil-Bazo I Toxic epidermal necrolysis related to pemetrexed and carboplatin with vitamin B12 and folic acid supplementation for advanced non-small cell lung cancer Onkologie 2009 32 580 584 10.1159/000232315 19816075

[B14] Bosch-Barrera J Montero A López-Picazo JM García-Foncillas J Ferrer M Yuste JR Gil-Bazo I Adult onset Still's disease after first cycle of pemetrexed and gemcitabine for non-small cell lung cancer Lung Cancer 2009 64 124 126 10.1016/j.lungcan.2008.09.013 19008012

[B15] Michels J Spano JP Brocheriou I Deray G Khayat D Izzedine H Acute tubular necrosis and interstitial nephritis during Pemetrexed therapy Case Rep Oncol 2009 2 53 56 10.1159/000208377 20740145 PMC2918829

[B16] Lurje G Manegold PC Ning Y Pohl A Zhang W Lenz HJ Thymidylate synthase gene variations: predictive and prognostic markers Mol Cancer Ther 2009 8 1000 1007 19383851 10.1158/1535-7163.MCT-08-0219

[B17] Salgado J Zabalegui N Gil C Monreal I Rodríguez J García-Foncillas J Polymorphisms in the thymidylate synthase and dihydropyrimidine dehydrogenase genes predict response and toxicity to capecitabine-raltitrexed in colorectal cancer Oncol Rep 2007 17 325 328 17203168

[B18] Bosch-Barrera J García-Franco C Guillén-Grima F Moreno-Jiménez M López-Picazo JM Gúrpide A Pérez-Gracia JL Aristu J Torre W García-Foncillas J Gil-Bazo I The multimodal management of locally advanced N2 non-small cell lung cancer: is there a role for surgical resection? A single institution's experience Clin Transl Oncol 2012 14 835 841 10.1007/s12094-012-0874-3 22855163

[B19] Takezawa K Okamoto I Okamoto W Takeda M Sakai K Tsukioka S Kuwata K Yamaguchi H Nishio K Nakagawa K Thymidylate synthase as a determinant of pemetrexed sensitivity in non-small cell lung cancer Br J Cancer 2011 104 1594 1601 10.1038/bjc.2011.129 21487406 PMC3101907

[B20] Shintani Y Ohta M Hirabayashi H Tanaka H Iuchi K Nakagawa K Maeda H Kido T Miyoshi S Matsuda H New prognostic indicator for non-small-cell lung cancer, quantitation of thymidylate synthase by real-time reverse transcription polymerase chain reaction Int J Cancer 2003 104 790 795 10.1002/ijc.11014 12640689

[B21] Hu Q Li X Su C Chen X Gao G Zhang J Zhao Y Li J Zhou C Correlation between thymidylate synthase gene polymorphisms and efficacy of pemetrexed in advanced non-small cell lung cancer Exp Ther Med 2012 4 1010 1016 23226765 10.3892/etm.2012.730 PMC3494125

[B22] Shi Q Zhang Z Neumann AS Li G Spitz MR Wei Q Case–control analysis of thymidylate synthase polymorphisms and risk of lung cancer Carcinogenesis 2005 26 649 656 15579479 10.1093/carcin/bgh351

[B23] Wang X Wang Y Wang Y Cheng J Wang Y Ha M Association of thymidylate synthase gene 3′-untranslated region polymorphism with sensitivity of non-small cell lung cancer to pemetrexed treatment: TS gene polymorphism and pemetrexed sensitivity in NSCLC J Biomed Sci 2013 20 5 10.1186/1423-0127-20-5 23350714 PMC3577430

[B24] Giovannetti E Lemos C Tekle C Smid K Nannizzi S Rodriguez JA Ricciardi S Danesi R Giaccone G Peters GJ Molecular mechanisms underlying the synergistic interaction of erlotinib, an epidermal growth factor receptor tyrosine kinase inhibitor, with the multitargeted antifolate pemetrexed in non-small-cell lung cancer cells Mol Pharmacol 2008 73 1290 1300 10.1124/mol.107.042382 18187583

